# Lifetime occupation is not associated with radiographic osteoarthritis of the first metatarsophalangeal joint in a cohort study of UK women

**DOI:** 10.1186/s13047-020-00429-5

**Published:** 2020-10-01

**Authors:** L. Cherry, L. Gates, N. K. Arden, C. J. Bowen

**Affiliations:** 1 School of Health Sciences, University of Southampton, Building 67, University Road, Southampton, SO17 1BJ UK; 2 Department of Podiatry, Solent NHS Trust, Southampton, UK; 3 Nuffield Department of Orthopaedics, Rheumatology and Musculoskeletal Sciences, University of Oxford, Oxford, UK; 4 Centre for Sport, Exercise and Osteoarthritis Research Versus Arthritis, Southampton, UK

**Keywords:** Occupation, Osteoarthritis, Foot, Feet, Metatarsophalangeal joint

## Abstract

**Objective:**

The study aim was to determine whether lifetime occupation was associated with the presence of radiographic osteoarthritis (ROA) of the first metatarsophalangeal joint (MTPJ) in women.

**Method:**

Data were collected from the prospective, population-based Chingford 1000 Women study. This cohort of women, aged 45–64 years at inception, was established in 1989 from a single general practice in Chingford, UK. Data has subsequently been collected repeatedly. Data from baseline, year six and year ten was used for the purposes of this cross-sectional study.

The primary outcome was the presence of dorsal view ROA of the first MTPJ. The main exposure was lifetime occupation, categorised according to levels of occupation previously defined via international consensus: 1. Sedentary, 2. Light, 3. Light manual, 4. Heavy manual. Logistic regression analyses were conducted to quantify the relationship between lifetime occupation type and the presence of ROA of the first MTPJ, adjusting for age, body mass index and lifetime high-heeled footwear use as potential interactive variables for each decade.

**Results:**

Data for 209 women were included within this study. The mean (SD) age was 57 (±5.2) years. Predominant lifetime occupation was reported as sedentary by 51.7%, as light by 0%, as light manual by 33.5% and as heavy manual by 14.8% of participants. There were no statistical associations between lifetime occupation type and the presence of ROA of the first MTPJ in either the unadjusted (OR = 0.99, CI = 0.78–1.26,*P* = 0.96) partially adjusted (for age and BMI; OR = 1.00, CI = 0.78–1.29, *P* = 0.99) or fully adjusted models (for age, BMI and lifetime high heel footwear use for each decade of working life (OR = 1.02, CI = 0.79–1.31, *P* = 0.91); high-heel footwear use up to 20s (OR = 0.83, CI = 0.71–1.31, *P* = 0.83); high-heel footwear use in 20–30s (OR = 1.00, CI = 0.75–1.3, *P* = 0.98); high-heel footwear use in 30–40s (OR = 1.00, CI = 0.70–1.42, *P* = 0.99); high-heel footwear use in 40–50s (OR = 0.90, CI = 0.58–1.40, *P* = 0.65); high-heel footwear use in 50s (OR = 0.63,CI = 0.36–1.09, *P* = 0.10).

**Conclusions:**

The findings suggest that lifetime occupation is not associated with the presence of ROA of the fist metatarsophalangeal joint. There does not appear to be any interactive effect between lifetime occupation, lifetime high-heel footwear use, age or BMI and ROA of the first MTPJ. In later life a positive trend towards increased ROA in those who reported lifetime high-heel footwear use was noted and this may be worthy of further research.

## Background

Osteoarthritis (OA) is a chronic and progressive joint disease, typified by degeneration of cartilage and excessive bone marginal growth, that is considered the most globally prevalent chronic joint disease [1]. The societal and personal burden of OA is significant, and the lower limb burden is particularly great with knee and hip OA ranked as the 11th highest contributor to global disability [2]. Previous research has shown a high prevalence of radiographic foot OA of between 17 and 33% of the population [3, 4] and that much like the hip or knee, this may be symptomatic and associated with reduced physical activity and poorer health related quality of life [5–7]. Recent evidence suggests that within the foot, radiographic OA (ROA) once established is non-modifiable [8], making it increasingly important to explore potentially modifiable causes for its onset. However, effective demonstration of risk factors for onset and progression of foot ROA requires prospective longitudinal investigation of a large cohort of people with notation of incident ROA over time [9]; at the time of writing there are limited cohorts with such data available. Thus, beliefs about the potential causes and risks for foot ROA have been largely theoretically driven [4]. However, a unique longitudinal dataset established in 1989 with women based in Chingford UK, can be drawn upon to provide new data about lifetime occupation and the prevalence of foot ROA in later life.

It has long been hypothesised that the mechanical loading of joints associated with certain occupations may contribute to the development of ROA [10] and we hypothesise that this may be evident in the foot. This is theorised based upon reported evidence in other lower limb joints; for example, increased risk of symptomatic radiographic knee OA is apparent in miners, floor layers and carpenters [11, 12]. These are occupations that are likely to involve knee bending and possibly heavy lifting. In an extrapolation of this theory, it is possible that the first metatarsophalangeal joint (MTPJ) is at increased risk for the development of ROA because it is functionally pivotal to both weight bearing loading (e.g. bending) and sagittal plane progression during walking [13].

Occupational activities that physically load the foot – notably, heavy and manual activity for substantial parts of the working day, could contribute to the potential mechanical loading of the first MTPJ and thus the potential for the development and progression of ROA. Thus, it is important to determine whether there is an association between level of occupation and ROA of the first MTPJ.

It is also noteworthy, that unlike the hip or knee, the foot is also subject to the effect of footwear, which could be related to occupation (e.g. high-heeled shoe use as part of smart apparel). Thus, footwear should also be considered as a potential interacting variable, alongside age and BMI, in tests of association between occupation and ROA of the first MTPJ. The main aim of this study was therefore to determine if lifetime occupation is associated with RAO of the first MTPJ in women. Secondary analyses aimed to investigate the potential interactive effect of lifetime high-heel footwear use, age and BMI on this relationship, which have been suggested by previous authors as possible contributing factors [14].

## Methods

### Aim, study design, setting

This study was designed to investigate the relationship between predominant lifetime occupation and ROA of the first MTPJ in a population-based cohort of middle to older age women in the UK. A secondary aim was to determine the potential interactive effect of lifetime high-heel footwear use within a logistic regression model with ROA as the primary outcome variable and lifetime occupation as a key exposure variable.

The study objectives were to 1. Determine whether lifetime occupation is associated with ROA of the first MTPJ, 2. To adjust the logistic regression model for age, BMI and lifetime high-heel footwear use for each decade of working age, 3. To adjust the logistic regression model for lifetime high-heel footwear use, 4. To complete ancillary analyses to confirm the absence of interactions between exposure variables and the primary outcome (ROA) which may artificially alter the results of the logistic regression model.

The wider study was longitudinal and observational in design, although data is used in cross-section for the purposes of this study, allowing for robust analysis of associated exposures (e.g. lifetime occupation) against the primary outcome of interest (ROA of the first MTPJ).

Data were collected from the ongoing, prospective, population-based Chingford 1000 Women cohort study via questionnaires or/or clinical visits [15]. This cohort of women, aged 45–64 years at inception, was originally established in 1989 from a single general practice in Chingford, UK, to explore determinants of osteoarthritis and osteoporosis progression. Data has subsequently been collected near annually creating an important epidemiological resource and one of the few such cohorts to include foot health data. Occupation and age data was used from baseline, x-ray and BMI from year 6 and lifetime footwear from year 10.

### Ethics, consent and permissions

Ethical permission to undertake this work was granted by the Waltham Forest and Redbridge local research ethics committee (reference: LREC R&WF 96); the study was sponsored by Whipps Cross Hospital Research and Development Unit. All participants gave informed consent in writing for their data to be collected and used for research purposes.

### Participants

The original study inclusion criteria were for women, aged 45–64 years, registered with a single general practice in the Chingford area of Essex, UK. Of the original 1003 participants at baseline, 872 attended year 6. Of these 209 had complete data on foot-x-ray and occupational exposure and were included in the current analysis.

### Primary outcome

The primary outcome was the presence or absence of ROA of either first MTPJ, completed at year six follow-up; as determined using a modified, single plane, La Trobe foot atlas, positively documented if grades of ≥2 for joint space narrowing or osteophyte formation were observed for either foot, and was derived from semi-weight-bearing, dorsoplantar view, foot radiographic films [16]. All radiographs were scored by a single rater; the reliability of scoring foot radiographs using this method has been reported previously as moderate (k = 0.51) to substantial (k = 0.61) for each domain [17].

### Explanatory variables

The main exposure variable was predominant lifetime occupation recorded at baseline, which was captured with the following question and responses: “Previous occupation: Housewife; Retired; Higher Manager / professional; Skilled Manager / Teacher / Nurse; Admin / secretary / clerical; Skilled Manual; Unskilled manual; 9=cleaning / domestic?”. Occupation was categorised according to levels of activity previously defined via international consensus study, resulting in the assignment of each original group into one of four levels: 1. sedentary, 2. light, 3. light manual, 4. heavy manual [18] (see Table 1 for further detail).

**Table 1:** Occupation categorisation

Original variable	Occupation level re-classification
Higher manager/ profession	Sedentary
Admin / secretary / clerical	Sedentary
Housewife	Light manual
Skilled manager / teacher / nurse	Light manual
Cleaning / domestic	Light manual
Skilled manual	Heavy manual
Unskilled manual	Heavy manual
Retired	Missing

Other explanatory variables included age, body mass index and lifetime high-heeled footwear use. In line with radiographs BMI and age were also collected at year six. For footwear the question “Heel height worn at age xx” for each of the following decades of life was asked: under 20, 20–30, 30–40, 40–50, 50+. “Heel height worn at age xx?”. Footwear was dichotomised to low or high-heeled shoe use; a heeled shoe was defined as having a heel height of greater than two or more inches. This cut off was chosen based on that defined in the question proceeding these, which asked “‘Ever worn shoes with heels 2 inches high or more?”

Predominant heeled shoe use was defined as high if worn for eight or more hours per week on average over each decade of working age (under 20 years, 20–30 years, 30–40 years, 40–50 years, 50+ years). Eight or more hours was selected to represent a period of one working day of use per week; however, heel use was not restricted to whilst in work and could have related to occupation or social activity. Lifetime heeled shoe use was defined as high if the predominant footwear style was heeled and the duration of use was high in three or more of the five decades evaluated. Reported footwear data were recorded at year ten follow-up.

### Statistical analysis

Descriptive data for demographic characteristics were calculated and presented as means and standard deviations or frequencies and percentages depending upon data measurement scale.

All available data were used in logistic regression analyses to explore associations between predominant lifetime occupation and radiographic foot OA. Results are presented as odds ratios (OR) with associated 95% confidence intervals (95% CI). Statistical significance was defined at the 5% level and all analyses were undertaken using Stata 14 (StataCorp. 2015. *Stata Statistical Software*. College Station, TX: StataCorp LP).

Univariable logistic regression analysis was conducted to quantify the relationship between lifetime occupation type and the presence of ROA of the first MTPJ. Adjustments were made in a multivariable model for age, BMI and lifetime high-heel footwear use for each decade or as an expression of overall working lifetime. Ancillary logistic regression analyses to confirm the absence of interaction between secondary exposure variables (age, BMI, footwear) and the primary outcome (ROA) which may artificially alter the results of the primary predictive model were completed.

## Results

**Fig. 1:**
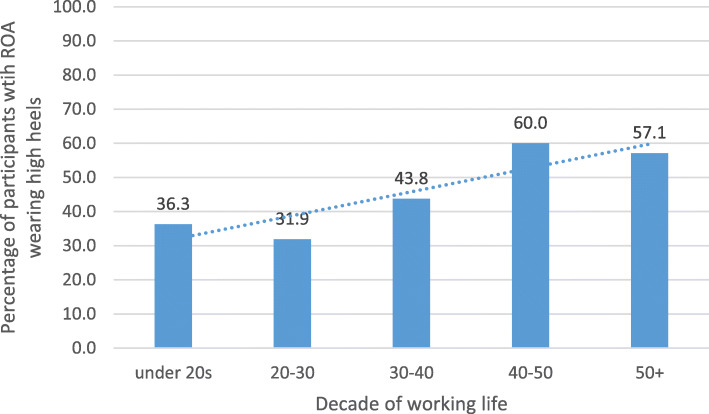
Percentage of people with ROA of the first MTPJ predominantly wearing high-heeled shoes in each decade of working life

A summary of participant demographics is included in Table 2. A summary of analysis results is included in Table 3. Overall, most participants reported a ‘sedentary’ lifetime occupation type (51.7%) with only 14.8% reporting to have had a ‘heavy manual’ lifetime occupation type. ROA of the first MTPJ was noted as present in 34.9% (*n* = 73) of participants; 15.1% (*n* = 11) of whom also reported a ‘heavy manual’ lifetime occupation state, 32.9% (*n* = 24) reported light manual and 52.1% (*n* = 38) reported sedentary The percentage of people with ROA of the first MTPJ predominantly wearing high-heeled shoes in each decade of working life is illustrated in Fig. 1.

**Table 2:** Cohort demographic and variable characteristics

Variable		Outcome n (%)
Radiographic OA (present/absent); present %		73/136 (34.9%)
Age (years); mean (±SD)		57.01 (5.23)
BMI (kg/m^2^); mean (±SD)		26.12 (4.12)
Predominant lifetime occupation; n (%)	(1) Sedentary	108 (51.7%)
	(2) Light	0 (0%)
	(3) Light manual	70 (33.5%)
	(4) Heavy manual	31 (14.8%)
Predominant heel height worn under 20 years of age (high/low); %		91/156 (58.33%)
Predominant heel height worn at 20–30 years of age (high/low); %		69/161 (42.86%)
Predominant heel height worn at 30–40 years of age (high/low); %		32/128 (25.00%)
Predominant heel height worn at 40–50 years of age (high/low); %		15/88 (17.05%)
Predominant heel height worn over 50 years of age (high/low); %		7/64 (10.94%)
Predominant lifetime high heel use (yes/no); %		7/209 (3.35%)

**Table 3:** Results of logistic regression analyses with ROA of the first MTPJ as the primary outcome

Predictor variable	Presence of ROA (R^**2**^)	Significance (***p***-value)	95% Confidence Interval (lower-upper bound)	OR
Lifetime occupation (LO)	0.000	0.963	0.78–0.26	0.99
LO adjusted for age and BMI	0.015	0.993	0.78–1.29	1.00
LO adjusted for age, BMI, and predominant heel height worn under 20 years of age	0.029	0.829	0.71–1.31	0.83
LO adjusted for age, BMI, and predominant heel height worn at 20–30 years of age	0.025	0.976	0.75–1.30	1.00
LO adjusted for age, BMI, and predominant heel height worn at 30–40 years of age	0.052	0.999	0.70–1.42	1.00
LO adjusted for age, BMI, and predominant heel height worn at 40–50 years of age	0.084	0.650	0.58–1.40	0.90
LO adjusted for age, BMI, and predominant heel height worn over 50 years of age	0.102	0.10	0.36–1.09	0.63
LO adjusted for age, BMI, and predominant lifetime high heel use	0.016	0.66	0.31–6.50	1.41

There were no statistically significant associations between lifetime occupation type and the presence of ROA of the first MTPJ in either the unadjusted (OR = 0.99,CI = 0.78–1.26, *P* = 0.96,) partially adjusted (for age and BMI; (OR = 1.00, CI = 0.78–1.29, *P* = 0.99) or fully adjusted models (for age, BMI and lifetime high-heel footwear use for each decade of working life (OR = 1.02, CI = 0.79–1.31, *P* = 0.91,); high-heel footwear use up to 20s (OR = 0.83, CI = 0.71–1.31, *P* = 0.83); high-heel footwear use in 20–30s (OR = 1.00, CI = 0.75–1.3, *P* = 0.98); high-heel footwear use in 30–40s (OR = 1.00 CI = 0.70–1.42, *P* = 0.99); high-heel footwear use in 40–50s (OR = 0.90, CI = 0.58–1.40,*P* = 0.65); high-heel footwear use in 50s (OR = 0.63, CI = 0.36–1.09, *P* = 0.10).

Ancillary analyses confirmed that age (OR = 1.05, CI = 1.00–1.12, *P* = 0.072) was independently associated with ROA of the first MTPJ, however neither BMI (OR = 1.03, CI = 0.96–1.10, *P* = 0.475) nor predominant lifetime high-heel use (OR = 1.41, CI = 0.31–6.50, *P* = 0.66) was independently significantly associated with ROA of the first MTPJ.

## Discussion

This study sought to explore whether lifetime occupation type (e.g. sedentary or heavy manual) could be related to OA of the foot, given that this has been previously demonstrated for the hip and knee [10, 19]. The findings of this study show for the first time that unlike other lower limb joints, lifetime occupation type does not appear to be significantly associated with radiographic first MTPJ OA in women. Whilst this is reassuring, occupation type remains an important factor to consider when delivering a clear public health and workforce policy message about lower limb OA. At present there is overwhelming evidence to suggest a need to develop tailored knee OA prevention strategies for industrial sectors due to the known relationship between manual occupations and knee OA; Our findings suggest this is not the case for first MTPJ OA in this generation of women. However, further work would be useful to determine if associations alter in different generations and between genders.

In a cross-sectional study to investigate the prevalence of symptomatic radiographic foot osteoarthritis in community dwelling older adults it was reported that 4.8% of those in managerial/administrative/professional occupational classes had first MTPJ OA, whilst 8.7% in intermediate occupational classes and 8.3% in routine and manual classes had first MTPJ OA [20]. In comparison the current study showed much higher frequencies of OA across levels of occupations, with 35% in Sedentary occupation, 34% in light manual and 35% in heavy manual having first MTPJ OA. These differences are likely to be due to the difference in definition of first MTPJ OA, however the generalisability of the sample within this study, recruited from a single geographical region within the UK, should also be considered as potentially noteworthy. Whilst Roddy et al. [20] reported symptomatic radiographic OA the current study reported specifically radiographic OA. There is a known discordance in the frequency of these two definitions of OA, with lower frequencies seen in symptomatic OA versus radiographic OA [21]. Also, the current study was a female only population, to note Roddy et al. [20] report a higher percentage of women with first MTPJ OA than men. Lastly the methods of occupation categorisation were different, in the current study levels of occupation were based on an original list of 8 occupation (Housewife; Retired; Higher Manager / professional; Skilled Manager / Teacher / Nurse; Admin / secretary / clerical; Skilled Manual; Unskilled manual; cleaning / domestic). Occupation in Roddy et al’s [20] study is based on 3 categories of occupational class for which it is not clear which type of jobs may be included in intermediate and routine classes, therefore making it difficult to compare the within study differences in OA frequencies between both studies.

Although reassuring in one regard, the findings of this study highlight how the mechanisms that may be driving the development and progression of foot OA may differ to those for other joints such as the hip or knee and thus the foot should continue to be an independent focus of investigation.

BMI and age have known effects on the association between occupation and OA of the knee [22]. It was therefore pertinent to consider these variables as potential confounders in the current analysis. Unlike the findings of previous published work exploring risk factors for knee OA, including that from this Chingford population, [10, 22] no notable change with adjustment for age or BMI was noted. The lack of effect noted within this study may be due to a different kinetic strain associated with the use of the first MTPJ compared to the knee, where this effect was more readily demonstrated. In a previous study examining the prevalence of symptomatic foot osteoarthritis [20] first MTPJ OA was seen to increase with age in females, with prevalence of 7.9% in age 50–64: 9.4% in age 65–74: and 10.3% in age 75+. Further exploratory analysis in the current study also showed an increase in prevalence of radiographic first MTPJ OA with age, however much higher prevalence of radiographic first MTPJ OA in similar age categories compared to Roddy et al. [20]; 33.7% prevalence in ages 49–64 and 45.0% in ages 65–74. Again, this difference is likely due the differences between symptomatic OA and radiographic OA.

Interestingly, when interrogating the data for the potential interactive/confounding effect of footwear, there was an increased frequency of first MTPJ OA observed in those wearing high-heels in older decades versus younger decades. However, as this was not a primary objective of the study and the sample size is small for each group; it is not possible to draw any conclusive statements about risk or causality. However, this trend may be worthy of further investigation, particularly as footwear may be a modifiable risk factor for foot ROA and key public health messages could arise from such work.

### Limitations

A limitation of this study is the inherent gender bias within the observed cohort – by nature of the design of this unique longitudinal cohort any inference is limited to women. As discussed previously, as the clinical and research community’s understanding of the pathogenesis of OA has progressed, there is increasing awareness that there could be key gender differences [23]. A recent meta-analysis by Sun et al. [24] has demonstrated that a consistent positive association between heavy physical workload and hip OA was observed only among male participants. It is important therefore to assert that it remains possible that lifetime occupation in men could be significantly associated with ROA of the first MTPJ. Further, this study is limited by exploration of only a few possible variables, based upon available data; exploration of other clinical or functional characteristics made possible with the advancement of modern technology (e.g. alternate imaging methods) and clinical approaches could yield interesting findings.

The method of defining lifetime occupation is arguably crude and lacks the sensitivity of an in-depth prospective study. There is potential for recall bias and underestimation of occupational load may be associated with household work for example gardening, cleaning, or laundry. Previous research suggests that it is more likely that occupational load is underestimated, and therefore it is possible that some diluting of potential associations has occurred with the majority reporting to be sedentary. Also, as a power calculation was not feasible, we cannot ensure that findings were not due to a type 2 statistical error.

## Conclusion

The findings suggest that lifetime occupation is not associated with the presence of ROA of the first metatarsophalangeal joint. There does not appear to be any interactive effect between lifetime occupation, high-heeled footwear, age or BMI and ROA of the first MTPJ. However, in those reporting use of high-heels there was a trend for increasing presence of first MTPJ ROA in later versus earlier life decades of wear and this may be worthy of further research.

## Data Availability

The data can be made available upon reasonable request.

## Abbreviations

BMI: Body Mass Index
MTPJ: Metatarsophalangeal joint
OA: osteoarthritis
ROA: Radiographic osteoarthritis
